# Diffusion tensor imaging of formalin fixed infarcted porcine hearts

**DOI:** 10.1186/1532-429X-15-S1-E103

**Published:** 2013-01-30

**Authors:** Ria Mazumder, Seongjin Choi, Brian Raterman, Bradley D Clymer, Arunark Kolipaka, Richard D White

**Affiliations:** 1 Department of Electrical and Computer Engineering, The Ohio State University, Columbus, OH, USA; 2 Department of Radiology, The Ohio State University Wexner Medical Center, Columbus, OH, USA; 3 Department of Internal Medicine, Division of Cardiology, The Ohio State University Wexner Medical Center, Columbus, OH, USA

## Background

Diffusion is the random motion exhibited by molecules as a result of thermal agitation. In biological tissues the random motion of water molecules is anisotropic since they are restricted by the tissue structure. The application of diffusion tensor imaging (DTI) makes it possible to quantify the amount of diffusion in tissues. Further processing allows a 3D visualization of the fiber architecture by tracking the fiber trajectories within a tissue. Experimental evidence has shown that fiber architecture in the myocardium changes with the onset of myocardial infarction [1]. Furthermore, the myocardium undergoes remodeling as the infarction progresses over time. The aim of this study is to evaluate the remodeling of the fiber architecture in an infarcted porcine heart.

## Methods

**Figure 1:**
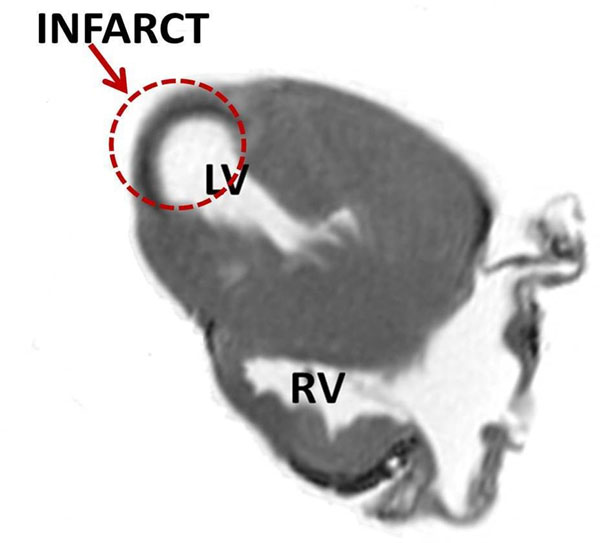
Magnitude image of the infarcted porcine heart, showing the infarcted region near the apex.

Ex-vivo DTI was performed on four infarcted porcine hearts on a 3T MRI scanner (Tim Trio, Siemens Healthcare). Infarcts were created in the apex region (Figure 1) by occluding the left anterior descending coronary artery, using balloon catheter. After 22 days, the hearts were dissected and formalin fixed for 6 months. A standard diffusion-weighted (DW) echo planar imaging (EPI) pulse sequence was used to acquire multi-slice short axis view of the heart. Imaging parameters included: diffusion encoding directions = 256; TE = 90 ms; TR = 6300,6600 ms; slice thickness = 2 mm; matrix = 128 x 128; FOV=256x256 mm^2^; b-values = 0,1000 s/mm^2^; slices = 40,42; resolution = 2 x 2 x 2 mm. The images were masked to segment the left ventricular myocardium (LVM). Explore DTI [2], was used to obtain a tensor map and then track the fibers using a deterministic algorithm. For this analysis, fractional anisotropy and the angle between the longest eigenvectors (V1) of the two successive voxels were set to 0.2 and 45 degrees respectively. The lower limit of the length of the fibers was varied from 2 mm to 30 mm to see the corresponding change in fiber tracts near the infarct in the LVM.

## Results

**Figure 2:**
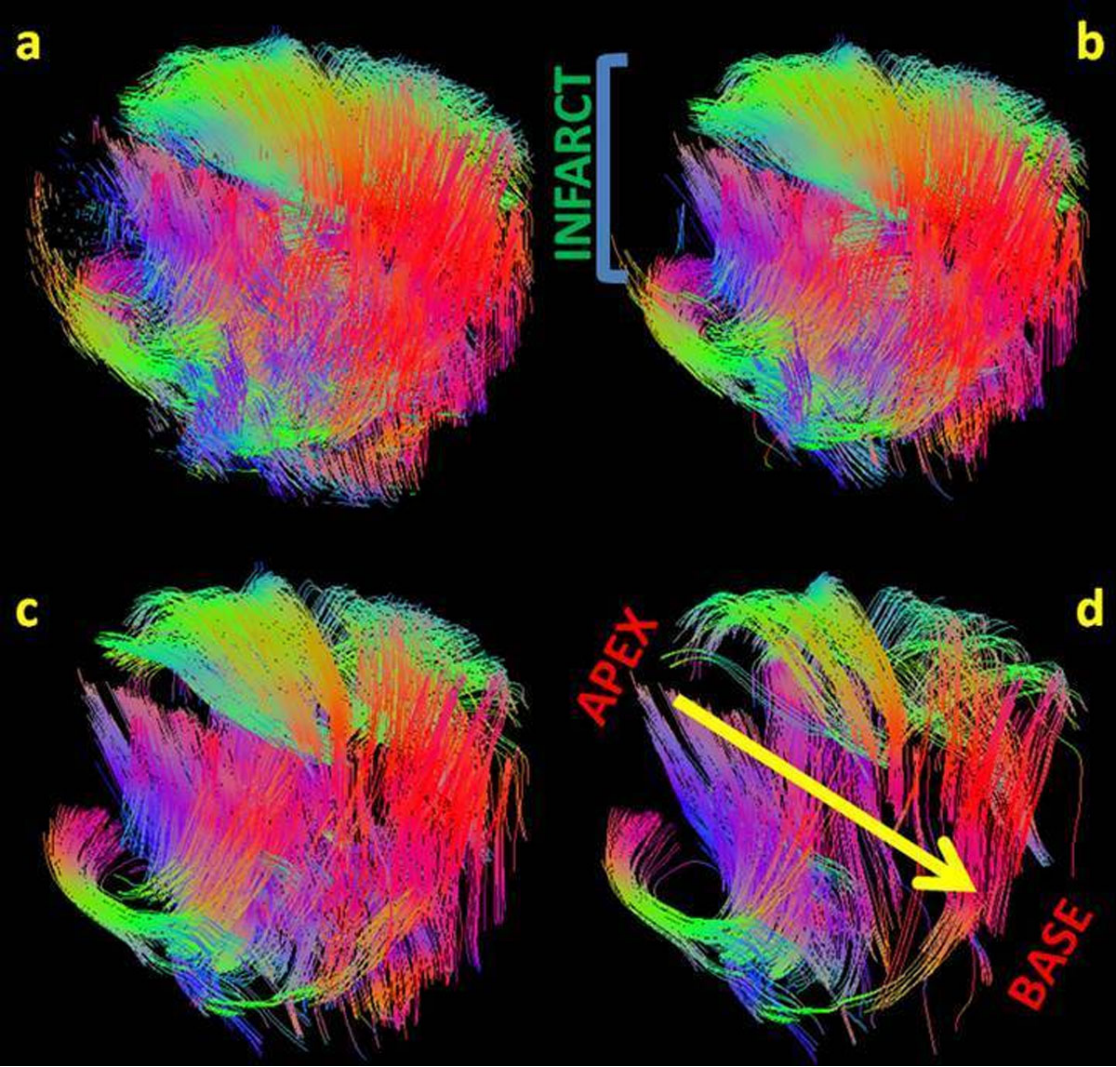
3D volumetric representation of fiber tracts in an infarcted porcine myocardium. a, b, c, d displays different ranges of fibers tracked by varying the lower limit of fiber length. a) 2-500 mm b) 10-500mm c) 20-500 mm and d) 30-500 mm. The direction of the yellow arrow on d indicates the progression from apex to base of the myocardium. The color code denotes the direction of fiber orientation; Green, red and blue corresponds to the x, y and z directions of the image respectively. At the apex, we observe disarrayed shorter fibers identifying the infarcted region and the fibers eventually vanish with increase in the lower limit of the fiber length from a to d.

Figure 1 shows the magnitude image displaying the infarcted region with thin myocardial wall. Figure 2 displays 3D visualization of the fiber tracts in the LVM in one of the excised hearts. From Figure 2(a) to 2(d) the lower limit of the fiber length was varied in the analysis to track the short disarrayed fibers near the infarct (the apex of the heart). Comparing Figure 2(a) (tracking length range: 2-500 mm) to 2(d) (tracking length range: 30-500 mm), we see that more fibers are tracked at the apex of the heart when shorter fiber lengths are included. Similar patterns were seen in the other three hearts. Based on these preliminary results we observe that the fibers around the infracted region are shorter in length and disarrayed compared to the rest of the LVM.

## Conclusions

The results demonstrated remodeling of fiber tissue structure in the LVM as a result of infarction.

However, more studies are warranted to confirm our analogy and establish the technique.

## Funding
